# Integration of risk variants from GWAS with SARS-CoV-2 RNA interactome prioritizes FUBP1 and RAB2A as risk genes for COVID-19

**DOI:** 10.1038/s41598-023-44705-3

**Published:** 2023-11-06

**Authors:** Weiwen Shi, Mengke Chen, Tingting Pan, Mengjie Chen, Yongjun Cheng, Yimei Hao, Sheng Chen, Yuanjia Tang

**Affiliations:** 1 Shanghai Institute of Rheumatology/Department of Rheumatology, Renji Hospital, Shanghai Jiao Tong University School of Medicine, Shanghai, China; 2 State Key Laboratory of Oncogenes and Related Genes, Shanghai Cancer Institute, Renji Hospital, Shanghai, China; 3 Department of Rheumatology, the First People’s Hospital of Wenling, Taizhou, China; 4 Key Laboratory of Tissue Microenvironment and Tumor, CAS Center for Excellence in Molecular Cell Science, Shanghai Institute of Nutrition and Health, Shanghai Institutes for Biological Sciences, University of Chinese Academy of Sciences, Chinese Academy of Sciences (CAS), Shanghai, China

**Keywords:** Genetics, Diseases

## Abstract

The role of host genetic factors in COVID-19 outcomes remains unclear despite various genome-wide association studies (GWAS). We annotate all significant variants and those variants in high LD (R^2^ > 0.8) from the COVID-19 host genetics initiative (HGI) and identify risk genes by recognizing genes intolerant nonsynonymous mutations in coding regions and genes associated with cis-expression quantitative trait loci (cis-eQTL) in non-coding regions. These genes are enriched in the immune response pathway and viral life cycle. It has been found that host RNA binding proteins (RBPs) participate in different phases of the SARS-CoV-2 life cycle. We collect 503 RBPs that interact with SARS-CoV-2 RNA concluded from in vitro studies. Combining risk genes from the HGI with RBPs, we identify two COVID-19 risk loci that regulate the expression levels of *FUBP1* and *RAB2A* in the lung. Due to the risk allele, COVID-19 patients show downregulation of *FUBP1* and upregulation of *RAB2A*. Using single-cell RNA sequencing data, we show that *FUBP1* and *RAB2A* are expressed in SARS-CoV-2-infected upper respiratory tract epithelial cells. We further identify NC_000001.11:g.77984833C>A and NC_000008.11:g.60559280T>C as functional variants by surveying allele-specific transcription factor sites and cis-regulatory elements and performing motif analysis. To sum up, our research, which associates human genetics with expression levels of RBPs, identifies *FUBP1* and *RAB2A* as two risk genes for COVID-19 and reveals the anti-viral role of FUBP1 and the pro-viral role of RAB2A in the infection of SARS-CoV-2.

## Introduction

The SARS-CoV-2 coronavirus is the pathogen causing the Coronavirus Disease 2019 (COVID-19) pandemic^1^, which has led to over 700 million infections, including 6 million deaths^2^. SARS-CoV-2 is an enveloped *Coronaviridae* family virus with a single-stranded, positive-sense (+) RNA genome^3^.

The genome-wide association study (GWAS) is a valuable tool for comprehending the genetic basis of complex traits and diseases related to the host. Many host-specific genetic variants associated with COVID-19 have already been identified using GWAS^4^. For example, genetic variants 12q24.13 mapped to OAS1 influence COVID-19 susceptibility and severity^5^. Despite the significant progress made in GWAS, there remains a major challenge in translating its findings to clinical application since numerous variants are located in non-coding regions^6^. Additionally, determining how genetic variants impact COVID-19 risk is still under investigation.

Cis expression quantitative trait loci (cis-eQTL) analysis effectively illustrates GWAS studies, especially in non-coding regions, because it associates genetic variants with gene expression. Studies combining GWAS statistics and cis-eQTLs have identified risk variants for COVID-19 that influence expression levels of susceptible genes^4^. For example, Horowitz et al. identified a genetic variant, rs190509934, which was a protective factor against SARS-CoV-2 infection and downregulated the expression of ACE2^7^. Moreover, it has been shown that functional non-coding variants can regulate gene expression levels in an allelic way by disrupting transcription factor (TF) binding sites^8^. It is helpful to associate host genetic variants of COVID-19 with the expression levels of genes to illustrate the result from GWAS.

SARS-CoV-2 highly depends on interactions with host cellular machinery to accomplish its viral life cycle and evade host defenses^9^. For example, the SARS-CoV-2 virus relies on the host’s protein synthesis machinery to produce essential viral proteins needed for replication. The replication of the SARS-CoV-2 genome involves an RNA-dependent RNA polymerase (RdRp) complex composed of non-structural protein 12 (NSP12) and two co-factors, NSP7 and NSP8, which bind to the viral RNA (vRNA) and initiate the synthesis of a complementary strand^10^. Additionally, SARS-CoV-2 encodes microRNA-like small RNAs, which target host functional genes to evade the host immune system^11,12^. On the other hand, the host cells activate anti-viral detection and defense mechanisms in response to SARS-CoV-2 infection. For example, RIG-I and MDA5 are cellular sensors that recognize SARS-CoV-2 RNA, and ZAP is an inhibitor of SARS-CoV-2 infection^13–15^.

RNA binding proteins (RBPs), which bind to RNA molecules and play an essential role in many aspects of biological processing, are one of the most crucial host cellular factors interacting with RNA viruses. RBPs participate in viral processing, RNA metabolism, RNA stability, and virus replication and translation^16–21^. Sufficient evidence has shown that host RBPs interacting with SARS-CoV-2 RNA (SARS-CoV-2 RNA interactome) participate in RNA metabolism, virus replication, anti-viral and pro-viral processes, and other pathways. For example, CNBP and LARP1 restrict SARS-CoV-2 replication, while IGF2BP1 stabilizes and augments the translation of SARS-CoV-2 RNA^22,23^. However, these studies utilizing RNA-centric methods to capture RBPs were not performed on cell lines derived from the primary lung. This limits their ability to accurately simulate natural human infections and establish an identical SARS-CoV-2 interactome that links host factors to COVID-19. Therefore, it is vital to associate host genetic risk variants of COVID-19 with expression levels of RBPs that interact with SARS-CoV-2 RNA and explore the mechanisms underlying them, providing strong evidence for the role of RBPs in COVID-19.

In this study, we focus on the SARS-CoV-2 RNA interactome in the lung to illustrate GWAS statics released by the COVID-19 Host Genetics Initiative (HGI) (release 7)^24^ using cis-eQTL mapping and aim to identify pathogenic variants that regulate the expression of RBPs to clarify the molecular mechanisms of how genetic variants influence the risk of COVID-19.

## Materials and methods

### Annotation of significant variants for COVID-19

GWAS data for COVID-19 were collected from the COVID-19 host genetics initiative (HGI) (https://www.covid19hg.org/)^24^. We used meta-analyzed COVID-19 data sets (phenotypes A2: critically ill vs. population, phenotypes B2: hospitalized COVID-19 vs. population, and phenotype C2: COVID-19 vs. population) from the eighth April 2022 release of COVID-19 Host Genetics Initiative including European ancestries provided by 23andMe (https://www.covid19hg.org/results/r7/). We used a p-value threshold of 5 × 10^−8^ to hit significant variants. PLINK v1.9 (http://pngu.mgh.harvard.edu/purcell/plink/)^25^ was used to find high LD variants (R^2^ > 0.8) from the European population of 1000G project GRCh38 phase 3 release data in Ensembl^26^. We then used the Variant Effect Predictor (VEP)^27^ to annotate all significant variants and those variants in high LD with them. The annotated variants were classified into coding and noncoding regions. Coding regions were further divided into synonymous and nonsynonymous mutations. We recognized genes intolerant nonsynonymous mutations in coding regions. The human lung eQTL data shown in this study were obtained from the Genotype-Tissue Expression (GTEx) Portal (dbGaP Accession phs000424.v8.p2)^28^ which contained data for 515 lung samples. We took the intersection between our hit non-coding variants and variants with eQTLs. We then identified genes regulated by the overlapping variants as eGENEs in the lung. Direct downloads of the cis-eQTL figures were made from the GTEx Portal.

### GO enrichment analysis

We conducted GO enrichment analysis of the union of RBP-encoding genes with nonsynonymous mutations and eGENEs using clusterProfiler v4.4.4 R package^29^. The treeplot function of Enrichplot v1.16.2 was used to cluster GO terms^30^.

### Collection of the human SARS-CoV-2 interactome

To identify total host RBPs interacting with SARS-CoV-2 RNA, we collected data from six studies on multiple cell lines using different approaches^31–36^. We manually excluded RBPs of other species and took a union of 503 human RBPs from these studies.

### LD plot of summary statistics for COVID-19

We used LDblockShow v1.40^37^ to calculate the linkage disequilibrium (R^2^) for the *FUBP1* locus (chr1: 77501100:78029110), *RAD50* locus (chr5: 132422500:132696349), and *RAB2A* locus (chr8: 60466936:60673644) from the European population of 1000G project GRCh38 phase 3 release data in Ensembl^26^. LDBlockshow was used to generate association statistics showing the − log10 p-value of COVID-19 risk variants and LD heatmaps showing the LD between COVID-19 variants in *FUBP1* and *RAB2A* loci.

### Single-cell RNA sequencing analysis

We obtained integrated Human Lung Cell Atlas (HLCA) single-cell RNA sequencing data via https://cellxgene.cziscience.com/collections/6f6d381a-7701-4781-935c-db10d30de293. Scanpy^38^ was used to analyze the data. We chose cells from COVID-19 patients. Sc. pl.umap(), sc.pl.dotplot(), and sc.pl.stacked_violin() functions were used to display the UMAP plot, dotplot, and violinplot, respectively.

We obtained single-cell RNA sequencing data from the nasopharynx of COVID-19 patients via the Single Cell Portal: https://singlecell.broadinstitute.org/single_cell/study/SCP1289/. Due to the small number of SARS-CoV-2 RNA^+^ cells (Supplementary Fig. 3c), we annotated the scRNA-seq data coarsely and combined ambient viral cells with SARS-CoV-2 RNA^−^ cells. Analysis was performed with Seurat v4.3.0^39^. We filtered cells by eliminating barcodes with fewer than 200 UMI, 150 unique genes, and greater than 50% mitochondrial reads. After log-normalization and scaling, we conducted principal component analysis (PCA) on the 2000 most variable genes for dimensionality reduction. We used Harmony v0.1.1^40^ to integrate the individual samples. We used the Jackstraw function to select the first 20 principal components to define a nearest neighbor graph and Uniform Manifold Approximation and Projection (UMAP) for dimension reduction. We then clustered cells using Louvain clustering (resolution = 0.5) following the use of clustree v0.5.0^41^. We used the FindAllMarkers function to calculate differentially expressed genes between each cluster and all other cells. Finally, we performed the cell-type annotation as previously described^42^, and marker genes are shown in Supplementary Fig. 3. DimPlot(), FeaturePlot(), DotPlot() and VlnPlot() function were used to create the UMAP plot, featureplot, dotplot and violinplot, respectively.

### Survey of allele-specific TF sites data

The survey of allele-specific TF sites was previously performed in ChIP-Seq data^43^. The authors identified nearly 270,000 allele-specific binding sites for TFs in that study via a meta-analysis of more than 7000 ChIP-Seq experiments. We queried all hit COVID-19 variants and those in high LD with the lead SNPs in ADASTRA database v5.1.2 (http://adastra.autosome.ru). Data were presented as the effect size and log2 of the p-value of reference and alternative allele, respectively.

### Identification of adult human lung cis-regulatory elements

From the ENCODE site^44^ (https://www.encodeproject.org), we downloaded epigenomic datasets of the human adult male lung (right lower lobe) in bigwig format. We then utilized the Integrative Genomics Viewer (IGV)^45^ based on GRCh38/hg38 for visualization. All files represent the fold change over the control for the assay presented. ENCFF928LLI (H3K4me1 ChIP-seq), ENCFF282VQS (H3K4me3 ChIP-seq), and ENCFF054VRQ (H3K27ac ChIP-seq) were the identifiers assigned to the three datasets.

### Transcription factor binding motif analysis.

We investigated functional COVID-19-associated variants for their potential effect on TF binding affinity using models from JASPAR2022 core collection^46^ and TFBSTools v1.34.0^47^. We defined a score threshold of 80% and a p-value of 5.00 × 10^−3^ to confirm the occurrence of the TF motif for any of the two alleles. DNA input of NC_000008.11:g.60559280T>C were ‘TTGCTGTGCAAGCCATTTCCCCGTTTCATGT’ and ‘TTGCTGTGCAAGCCACTCCCCGTTTCATGT’ and NC_000001.11:g.77984833C>A were ‘GTAGTTTCACACAAACTTTTCTTAGAATATC’ and ‘GTAGTTTCACACAAAATTTTCTTAGAATATC’. We used ggseqlogo v0.1^48^ to visualize the motif of target TFs, and NC_000001.11:g.77984833C>A was displayed in the negative strand.

### Correlation analysis between candidate TFs and RBPs

Gene expression data shown in correlation analysis were obtained from the genotype-tissue expression (GTEx) Portal (dbGaP Accession phs000424.v8.p2)^28^ containing data for 515 lung samples. We then used ggstatsplot v0.11.0 to display the correlation matrix and ggcorrplot v0.1.4 to visualize the scatterplot of candidate TFs and RBPs^49^.

## Results

### Identification of COVID-19 associated variants and risk genes

**Figure 1:**
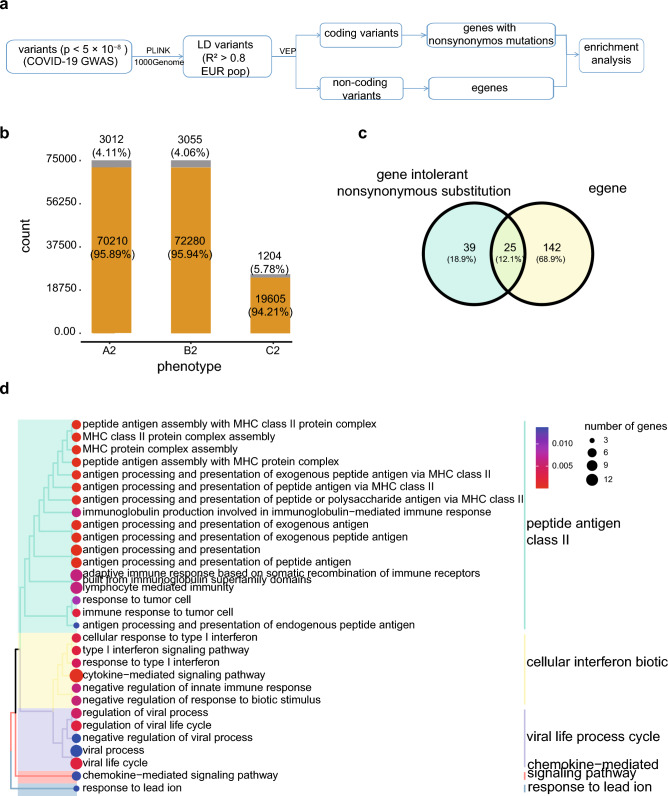
Atlas of potential risk genes for COVID-19 (**a**) Flowchart of the study design. (**b**) Distribution of genomic regional categories for COVID-19 variants. (**c**) Vennplot of genes intolerant nonsynonymous mutations and eGENEs. (**d**) Clustered GO terms of all potential risk genes for COVID-19.

Despite various GWAS mapping risk loci for COVID-19, the interpretation and mechanism underlying the study remain unknown. To gain a broader view of how genetic variants influence the susceptibility and severity of COVID-19, we took the location and consequences of risk variants into consideration (Fig. 1a). To interpret the results from HGI comprehensively, we found LD variants (R^2^ > 0.8) of significant SNPs (p < 5 × 10^−8^) and used the Variant Effect Predictor (VEP) to annotate all these variants (Supplementary Table 1) in European ancestry group (phenotype A2: critically ill vs. population, phenotype B2: hospitalized COVID-19 vs. population, and phenotype C2: COVID-19 vs. population). In these three phenotypes, about 4 % of variants were found in coding regions, while 96% were found in non-coding regions (Fig. 1b). In coding regions, we focused on genes intolerant nonsynonymous mutations. To further understand the potent target genes in non-coding regions, we surveyed eQTL in GTEx^28^ in lung tissue to identify genes whose expressions are associated with variants (eGENEs). Risk variants and variants with eQTLs were intersected, and the targeted genes regulated by eQTLs were defined as eGENEs. Overlapping genes intolerant nonsynonymous mutations, and overlapping eGENEs across three phenotypes were described in Supplementary Fig. 1a and b, respectively. The overlap of genes intolerant nonsynonymous mutations and eGENEs across three phenotypes was depicted in Fig. 1c. We then performed GO analysis on all target genes (genes intolerant nonsynonymous mutations and eGENEs) of COVID-19 to determine their overall biological function (Supplementary Table 2). Clustered GO terms were enriched in immune response pathways (peptide antigen, cellular interferon biotic), viral life cycle, and other signaling pathways (Fig. 1d), indicating that these risk genes were highly linked to COVID-19.

### SARS-CoV-2 interacting protein related risk loci for COVID-19

After identifying potential risk genes for COVID-19, it is crucial to associate host genetic factors with SARS-CoV-2 to illustrate the disease’s mechanism. As SARS-CoV-2 is an RNA virus, RBPs are one of the most direct ways the host interacts with the virus. Several studies have been conducted to examine the interaction between host proteins and RNA of SARS-CoV-2, to identify the host RBPs that directly bind SARS-CoV-2 RNA and furnish proof of the roles RBPs play in the infection process of SARS-CoV-2. In the previous six studies^31–36^ (Supplementary Table 3), 503 host RBPs interacting with SARS-CoV-2 RNA have been identified in multiple human infected cell lines using different RNA-centric cross-linking approaches (Supplementary Fig. 2a). As it is shown in Supplementary Fig. 2a, these in vitro results have shown differences in identifying the SARS-CoV-2 RNA interactome, possibly due to RNA-centric procedures and different cell contexts. GO analysis has showed that host RBPs participated in multiple steps of the SARS-CoV-2 life cycle (Supplementary Fig. 2b,c).

We surveyed the 503 RBPs to determine whether the risk genes described above function in interacting with SARS-CoV-2. We observed that risk genes RAD50 double strand break repair protein (*RAD50*), Far Upstream Element Binding Protein 1 (*FUBP1*), and RAB2A, member RAS oncogene family (*RAB2A*), were RBPs that interacted with SARS-CoV-2 RNA. We then identified risk variants that regulate the expression of RBPs from GWAS statistics, all located in non-coding regions (Table 1). We assumed that these polymorphic loci participate in COVID-19 by affecting the expression of RBPs.

**Table 1:** COVID-19 risk variants regulate the expression level of RBPs.

Chr	Variant	Risk allele	Genomic annotation	p-value			Locus gene	eGENE
				A2	B2	C2		
1	NC_000001.11:g.77501822T>A	A	Intron	7.64e-07	1.98e-08^a^	0.0019706	AK5	FUBP1
	NC_000001.11:g.77984833C>A	A	Intron	1.17e-06	3.41e-09^a^	0.00034365	DNAJB4	
5	NC_000005.10:g.132422622A>G	A	Intron	2.01e-08^a^	5.97e-07	0.0048754	IRF1-AS1	RAD50
	NC_000005.10:g.132423020T>C	T	Intron	2.01e-08^a^	3.50e-07	0.0013191		
	NC_000005.10:g.132424387A>G	A	Intron	3.41e-08^a^	6.69e-06	0.013389		
	NC_000005.10:g.132424726A>G	A	Intron	2.01e-08^a^	1.10e-06	0.0096258		
	NC_000005.10:g.132424758A>G	A	Intron	3.29e-08^a^	1.84e-06	0.0048866		
	NC_000005.10:g.132427482T>C	T	Intron	3.40e-08^a^	3.32e-07	0.0039825		
	NC_000005.10:g.132428411A>G	A	Intron	2.84e-08^a^	3.08e-07	0.0042891		
	NC_000005.10:g.132441275T>C	C	Intron	1.96e-08^a^	3.34e-08^a^	0.001593		
	NC_000005.10:g.132448315C>T	T	Intron	4.57e-08^a^	1.96e-08^a^	0.0021256		
	NC_000005.10:g.132453764G>A	A	Intron	2.58e-08^a^	3.76e-08^a^	0.003704		
	NC_000005.10:g.132457732dup	TG	Intron	6.33e-09^a^	2.04e-07	4.07e-05		
	NC_000005.10:g.132458098T>G	G	Intron	2.62e-08^a^	2.39e-08^a^	0.0036656		
	NC_000005.10:g.132459227dup	TC	Intron	1.27e-08^a^	1.99e-07	0.00043893		
	NC_000005.10:g.132462711C>G	G	Intron	3.28e-08^a^	5.98e-08^a^	0.0051042		
	NC_000005.10:g.132463037C>T	T	Intron	4.02e-08^a^	8.14e-08^a^	0.0055856		
	NC_000005.10:g.132463928G>A	A	Intron	3.98e-08^a^	9.48e-08^a^	0.0062497		
8	NC_000008.11:g.60515641C>T	T	Upstream	5.61e-06	4.81e-08^a^	3.89e-05	RAB2A	RAB2A

Phenotype A2: critically ill vs. population, Phenotype B2: hospitalized COVID-19 vs. population, and Phenotype C2: COVID-19 vs. population.

^a^p-value < 5 × 10^–8^.

**Figure 2:**
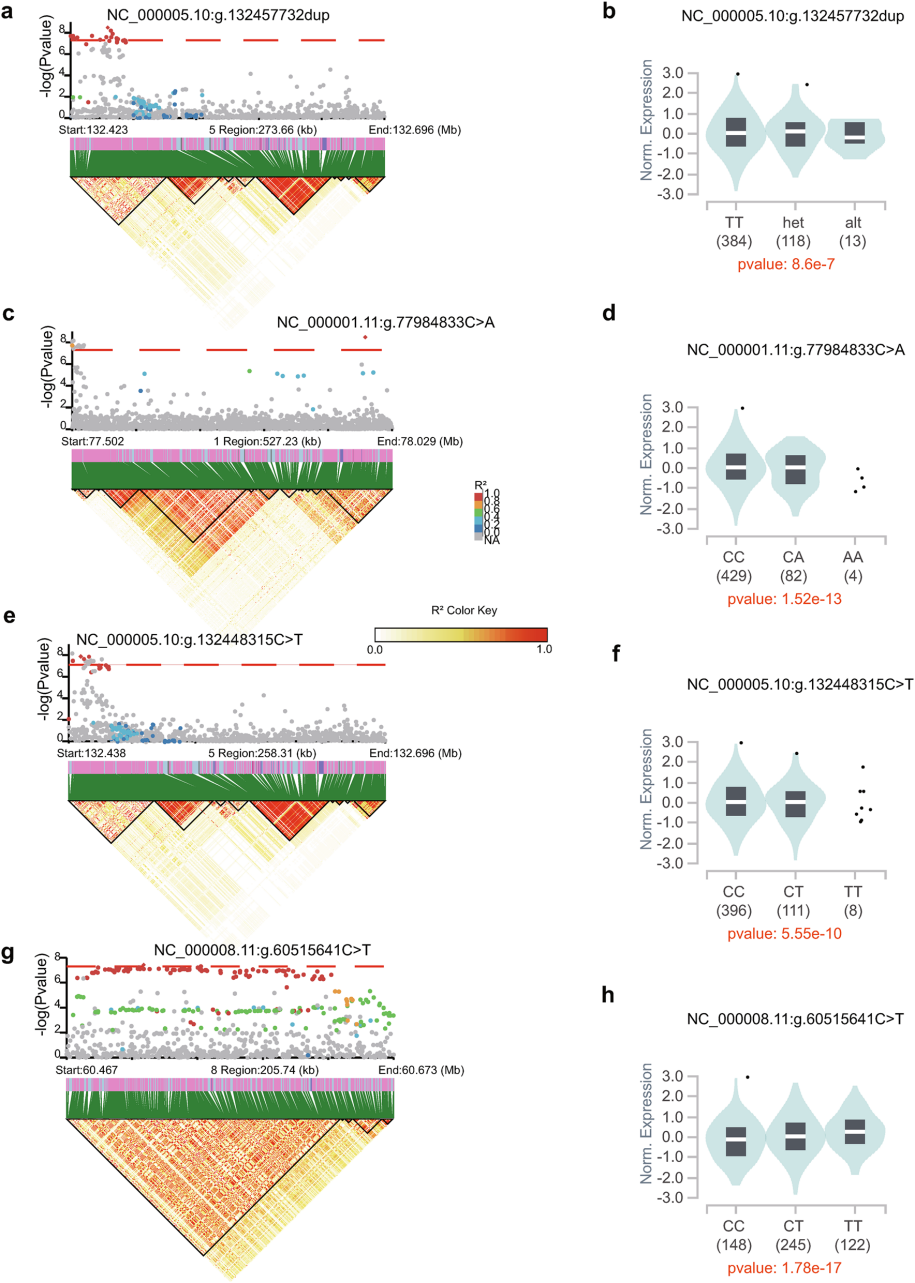
COVID-19 risk variants near the RBP loci regulate the expression of RAD50, *FUBP1,* and *RAB2A*. LD plot of (**a**) 273kb on 5q31.1 in A2 phenotype, and (**c**) 527kb regions on 1p31.1., (**e**) 258kb regions on 5q31.1, and (**g**) 205 kb regions on 8q12.1-8q12.2 in B2 phenotype. In the upper panel, the y-axis represents the −log10 of p-values for the GWAS meta-analysis from HGI, and the x-axis represents the chromosomal positions based on the GRCh38/hg38 assembly. The red dotted line indicates −log10 of the p-value threshold (5 × 10^-8^). Each dot represents a single nucleotide polymorphism (SNP) from the GWAS of COVID-19. The colored dots represent cis-eQTL variants. The colors indicate pairwise LD’s strength according to the R^2^ matrix from the 1000 Genome European population. The square dots represent the lead SNP. The lower panel shows an LD heatmap plot. Violin plots from the GTEx v8 human lung cis-eQTLs illustrate the number of samples in each genotype (adjusted p-values are shown below). (**b**) COVID-19 severity variant NC_000005.10:g.132457732dup is a cis-eQTL upregulating *RAD50*, (**d**) COVID-19 hospitalization variant NC_000001.11:g.77984833C>A is a cis-eQTL downregulating *FUBP1*, (**f**) COVID-19 hospitalization variant NC_000005.10:g.132448315C>T is a cis-eQTL upregulating *RAD50*, and (**h**) COVID-19 hospitalization variant NC_000008.11:g.60515641C>T is a cis-eQTL upregulating *RAB2A*.

Combining eGENEs and RBPS, we identified three loci, including *FUBP1* (chr. 1p31.1), *RAD50* (chr. 5q31.1), and *RAB2A* (chr. 8q12.1), where COVID-19 risk associated variants modified gene expression of RBPs in the lung tissue. We used LDBlockShow to create an LD plot^37^, using a calculated LD matrix from the 1000 Genomes European reference^50^ (accounting for 85% of cis-eQTL populations) (Fig. 2a,c,e,g).

In chromosome 1, we identified two genetic variants as cis-eQTLs associated with the eGENE *FUBP1* in the lung for hospitalization. NC_000001.11:g.77501822T>A is located in the AK5 locus downstream FUBP1, while NC_000001.11:g.77984833C>A is located upstream FUBP1. We found that risk allele carriers of the lead SNP NC_000001.11:g.77984833C>A had a significantly higher risk of worse disease outcomes than non-carriers (MAF = 0.1191, beta = 0.092186, p = 3.41 × 10^−9^). Fig. 2d shows that this risk allele was linked to a lower level of FUBP1 expression in the lung. These results demonstrate that NC_000001.11:g.77501822T in *FUBP1* is linked to a higher rate of SARS-CoV-2 hospitalization and downregulates the expression of *FUBP1* in the lung, which may limit FUBP1 anti-viral activity and thereby contribute to the development of the SARS-CoV-2 infection.

In chromosome 5, we identified sixteen and four genetic variants that increased the risk of severity and hospitalization of COVID-19, respectively. These variants were located within a region of strong LD and were cis-eQTLs associated with eGENE *RAD50*. GWAS analysis indicated that the lead SNP NC_000005.10:g.132457732dup (MAF = 0.1141, beta = 0.1129, p = 6.33 × 10^−9^) for the A2 phenotype and NC_000005.10:g.132448315C>T (MAF = 0.1129, beta = 0.081636, p = 1.96 × 10^−8^) for the B2 phenotype upregulated the expression of *RAD50* in the lung (Fig. 2b,f). These findings suggested that this locus is a risk factor for SARS-CoV-2 by promoting the expression of *RAD50* in the lung, highlighting the pro-viral role of RAD50 in SARS-CoV-2 infection.

In chromosome 8, we identified a genetic variant NC_000008.11:g.60515641C>T, an SNP located upstream the RAB2A locus, as a cis-eQTL associated with the eGENE RAB2A in the lung for hospitalization. We found that risk allele carriers of NC_000008.11:g.60515641C>T had a significantly higher risk of worse disease outcomes than non-carriers (MAF = 0.3814, beta = 0.053141, p = 4.81 × 10^−8^). Cis-eQTL data showed that this risk allele was associated with higher expression of RAB2A in the lung (Fig. 2h). These findings suggest that NC_000008.11:g.60515641C>T is a risk factor for SARS-CoV-2 hospitalization by promoting the expression of *RAB2A* in the lung, highlighting the pro-viral role of RAB2A in SARS-CoV-2 infection.

The results indicate that the risk allele NC_000005.10:g.132457732dup renders severity of COVID-19 through upregulating *RAD50*, and NC_000001.11:g.77984833C>A, NC_000005.10:g.132448315C>T, and NC_000008.11:g.60515641C>T contribute to the hospitalization of COVID-19 through downregulating *FUBP1* and upregulating *RAD50* and *RAB2A*, respectively.

### FUBP1 and RAB2A are expressed in the SARS-CoV-2-infected epithelial cells of nasopharynx in COVID-19 patients

After identifying risk variants for COVID-19 which regulate the expression level of RBPs, it is necessary to validate the expression of interested RBPs in SARS-CoV-2 targeted tissue and infected cells to provide evidence for the interaction between our interested RBPs and SARS-CoV-2 RNA due to heterogeneity in identifying the SARS-CoV-2 RNA interactome from different RNA-centric procedures and different cell contexts (Supplementary Fig. 2).

**Figure 3:**
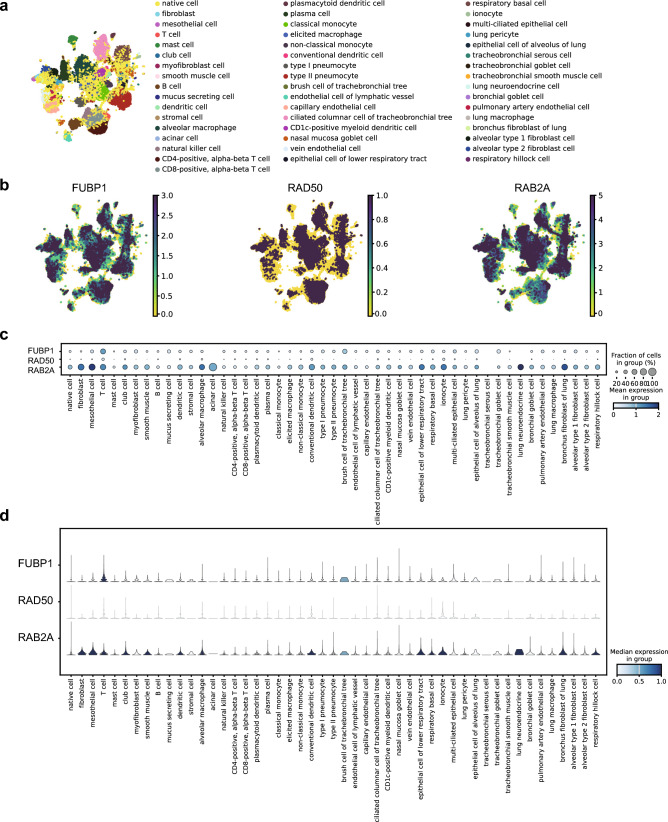
Expression of *FUBP1*, *RAD50*, and *RAB2A* in single-cell data in COVID-19 patients from HLCA. (**a**) UMAP of cell types of single-cell data in COVID-19 patients from HLCA. (**b**) The Feature plots showing the expression levels of *FUBP1*, *RAD50,* and *RAB2A* in COVID-19 patients. (**c**) Dotplot and (**d**) Violin plot showing the number of cells expressing *FUBP1*, *RAD50,* and *RAB2A* in COVID-19 patients.

To investigate the expression of our targeted RBPs, we first chose single-cell RNA sequencing (scRNA-seq) data of COVID-19 patients from the integrated Human Lung Cell Atlas (HLCA)^51^, a large-scale, cross-dataset scRNA-seq of the lung. The COVID-19 patients scRNA-seq data from HLCA included 60 samples and 49 cell types (Fig. 3a). We found that FUBP1 and RAB2A were expressed in multiple cell types, such as club cells, brush cell of the tracheobronchial tree, and epithelial cell of alveolus of lung while RAD50 was expressed lowly in almost all cell types (Fig. 3b–d).

**Figure 4:**
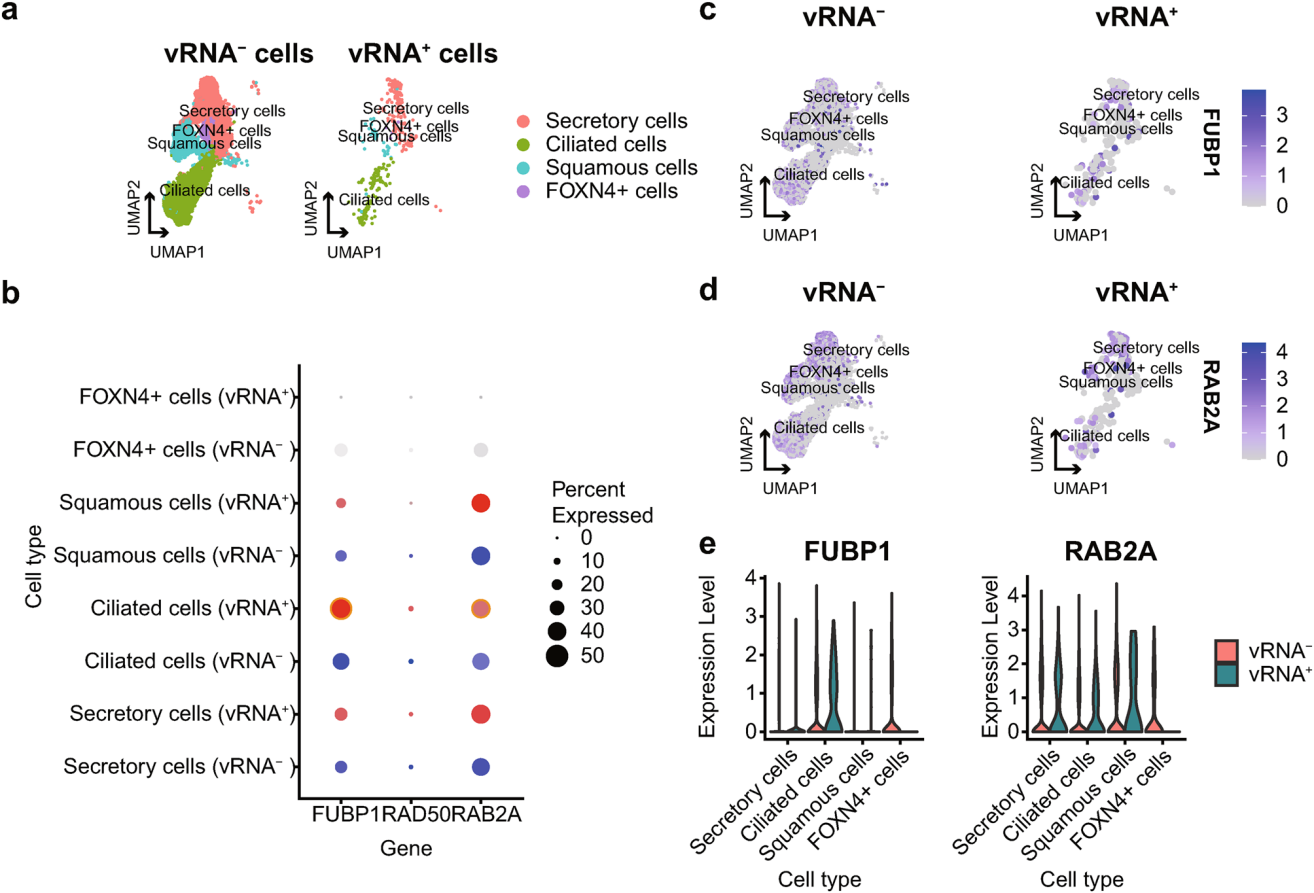
Expression of *FUBP1*, *RAD50*, and *RAB2A* in single-cell data in the nasopharynx. (**a**) UMAP of the top four epithelial cells with a high percentage of SARS-CoV-2 RNA^+^ (vRNA^+^) cells in the nasopharynx. (**b**) Dotplot showing the number of cells expressing *FUBP1*, *RAD50,* and *RAB2A* in both vRNA^+^ and SARS-CoV-2 RNA ^−^ (vRNA^−^) groups. The dots with yellow frames indicate expression levels of *FUBP1* and *RAB2A* in ciliated cells, which SARS-CoV-2 enters in airway epithelia immediately upon infection^54^. The feature plots show the expression levels of (**c**) *FUBP1* and (**d**) *RAB2A* in the four epithelial cells, which are grouped by SARS-CoV-2 RNA status. (**e**) The violin plot of *FUBP1* and *RAB2A* expressed in epithelial cells, grouped by SARS-CoV-2 RNA status.

To further examine the manifestation of targeted RBPs in cells that have been infected, we chose another scRNA-seq data from nasopharyngeal samples because SARS-CoV-2 infection primarily affects the upper respiratory tract^52^. We analyzed scRNA-seq data from COVID-19 patients reported by Ziegler CGK et al.^53^ and annotated cell types coarsely due to the limited number of cells identified as positive for SARS-CoV-2 RNA (vRNA^+^) (Supplementary Fig. 3b). Marker genes were shown in Supplementary Fig. 3a. We demonstrated the top four epithelial cells (secretory cells, ciliated cells, squamous cells and FOXN4^+^ cells) with a high percentage of vRNA^+^ cells (Supplementary Fig. 3c, Fig. 4a) to display the most possible SARS-CoV-2-infected epithelial cells. The results showed that *FUBP1* and *RAB2A* are expressed in several types of nasopharyngeal epithelial cells, including FOXN4^+^ cells, squamous cells, ciliated cells, and secretory cells, in the upper respiratory tract of COVID-19 patients, especially the vRNA^+^ group (Fig. 4c,d). Notably, *FUBP1* and *RAB2A* were highly expressed in vRNA^+^ ciliated cells (Fig. 4b), which were the primary target cells of SARS-CoV-2 upon infection^54^. Similar to the result from HLCA, RAD50 was low expressed in epithelial cells (Fig. 4b, Supplementary Fig. 3d).

These findings show that *FUBP1* and *RAB2A* are expressed in SARS-CoV-2-infected epithelial cells, which lays the foundation for the interaction between SARS-CoV-2 RNA and our targeted RBPs, indicating that FUBP1 and RAB2A may play essential roles in the infection of SARS-CoV-2.

### Functional variants that regulate the expression of FUBP1 and RAB2A

Since COVID-19 risk variants regulating RBPs are all in non-coding regions, it is challenging to identify the causal variants. Reports suggest that non-coding variants can potentially regulate the expression of target genes by either disrupting the underlying TF binding sites or altering the strength of regulatory regions^58,59^.

**Figure 5:**
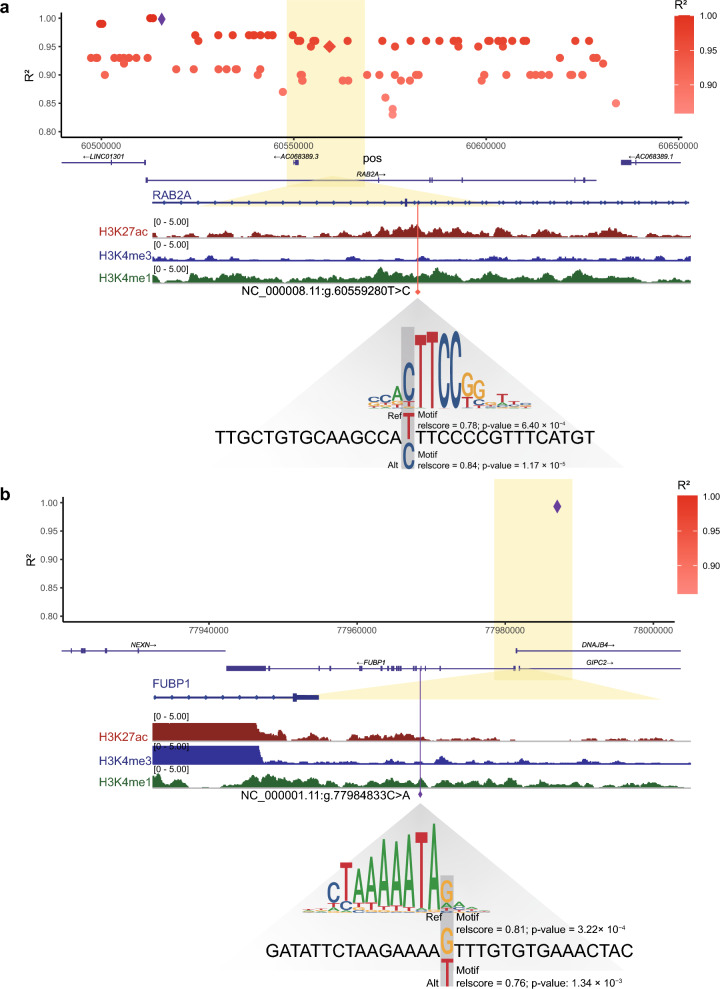
Epigenomic data and TF motif analysis to identify allele-specific functional variants that regulate the expression of *FUBP1* and *RBA2A*. LD plot showing R^2^ of variants in LD with the lead SNPs. The y-axis represents the R^2^ for variants in LD with the lead SNPs, and the x-axis represents the chromosomal positions based on the GRCh38/hg38 assembly. The lead SNPs are displayed as purple diamonds. The functional variants are displayed as red squares. The color gradient indicates pairwise LD’s strength (R^2^) according to 1000 Genomes European Reference. (**a**) NC_000008.11:g.60559280T>C is strongly linked with the lead SNP NC_000008.11:g.60515641C>T (R^2^ = 0.95), overlapping an activated lung enhancer within the *RAB2A* gene. Transcription factor motif analysis indicates that the COVID-19 variant NC_000008.11:g.60559280T>C increases the binding affinity of a motif for the ELF1. (**b**) NC_000001.11:g.77984833C>A is in lung enhancer upstream of the *FUBP1* gene. Transcription factor motif analysis indicates that the COVID-19 variant NC_000001.11:g.77984833C>A (negative strand) increases the binding affinity of a motif for the MEF2A.

To identify the likely causal variants and explore the genetic mechanisms driving COVID-19 hospitalization risk, we first surveyed the ADASTRA database v5.1.2^43,55^, which identified allele-specific TF binding at SNPs in Chromatin Immunoprecipitation Sequencing (ChIP-Seq) data. After analyzing all hit variants and those in high LD with the lead SNPs in the ADASTRA database v5.1.2^43,55^, we found that NC_000008.11:g.60559280T>C, which was highly linked with lead SNP NC_000008.11:g.60515641C>T mapped to *RAB2A*, is an allele-specific binding site for twelve TFs (Supplementary Table 4), which made us prefer NC_000008.11:g.60559280T>C as a functional variant. Using the scRNA-seq described above, we examined the expression of SPI1 and ELF1, which concordantly matched motif analysis and were more credible. It was observed that ELF1 exhibited a higher expression level (Supplementary Fig. 4a), leading us to recognize ELF1 as the effector TF, whose binding affinity was altered in NC_000008.11:g.60559280T>C (Fig. 5a). Correlation analysis showed that *ELF1* was positively associated with *RAB2A* (Supplementary Fig. 4e), which correlates with its transcriptional function^56^. We then used TFBStools^47^ to predict the binding affinity of ELF1 on the reference and alternative alleles of NC_000008.11:g.60559280T>C. The p-value changed from 6.40 × 10^−3^ (allele T) to 1.17 × 10^−3^ (allele C), so the binding affinity increased in the risk allele. Surveying epigenomic data in the lung, we found NC_000008.11:g.60559280T>C located in an activated lung enhancer region through epigenomic data (Fig. 5a), which demonstrated the transcriptional activity of NC_000008.11:g.60559280T>C. The result shows that the risk allele of NC_000008.11:g.60559280T>C, which is highly linked to NC_000008.11:g.60515641C>T, is connected to higher transcription levels of *RAB2A* and is believed to potentially strengthen the binding affinity towards ELF1, a transcriptional activator.

For variants mapped to *FUBP1*, which did not hit the ADASTRA database, we assumed that the variants were more likely functional if located within active lung CREs. We surveyed the regulatory regions in the lung and found NC_000001.11:g.77984833C>A located in the enhancer regions of *FUBP1* (Fig. 5b), so we identified this variant as the functional variant. We then performed TF analysis on NC_000001.11:g.77984833C>A by TFBStools^47^. We used all version models from JASPAR2022 core collection^46^ and set the variant ±15bp as input (detailed in the method). We then defined a score threshold of the TFs as 80% and p-value as 0.005 to confirm the occurrence of the TF motif for both reference and alternative alleles (Supplementary Table 5). We found 12 candidate TFs whose binding affinity changed at NC_000001.11:g.77984833C>A. We also investigated the expression of the candidate TFs in the scRNA-seq described above (Supplementary Fig. 4a). We then performed correlation analysis between these highly expressed TFs and FUBP1, and the activator *MEF2A*^57^ was positively associated with *FUBP1* (Supplementary Fig. 4f), which correlated with its transcriptional function, so we identified *MEF2A* as the effector TF. The transcription of *FUBP1* was anticipated to be lowered by the risk allele (A) of NC_000001.11:g.77984833C>A. The binding affinity of the transcriptional activator *MEF2A* was predicted to decrease at NC_000001.11:g.77984833C>A. The results show that activator MEF2A binds more weakly in the risk allele of NC_000001.11:g.77984833C>A, thus reducing the transcription of FUBP1 and leading to more hospitalizations.

Through the strategy mentioned above, we identify two functional variants that regulate the expression of *FUBP1* and *RAB2A* by changing TF binding affinity, respectively. NC_000008.11:g.60559280T>C, located in an activated enhancer region of *RAB2A*, upregulates *RAB2A* expression by increasing the affinity of the activator ELF1, while NC_000001.11:g.77984833C>A, located in the enhancer regions of *FUBP1*, downregulates *FUBP1* expression by decreasing the binding affinity of the activator *MEF2A*.

## Discussion

Genetic variants have been discovered to predispose COVID-19 patients to a more severe outcome, such as hospitalization. The interpretation of GWAS is vital for understanding the mechanisms underlying COVID-19. We annotated all variants and those variants in high LD (R^2^ >0.8) and classified them as coding and non-coding variants. We further identified risk genes from genes with nonsynonymous mutations and eGENEs in the lung. To investigate how these risk genes function in COVID-19, we took RBPs, which have been recognized to engage in multiple stages of the SARS-CoV-2 life cycle, into consideration and identified three RBP-related loci for COVID-19. Subsequently, we demonstrated the expression levels of *FUBP1*, *RAD50,* and *RAB2A* in epithelial cells infected with SARS-CoV-2 in scRNA-seq data and speculated that RAD50 might not function in the lung because of its low expression. We further chose an allele-specific strategy and identified NC_000001.11:g.77984833C>A and NC_000008.11:g.60559280T>C as functional variants. TF binding affinity changed at these two variants contributed to the hospitalization of COVID-19 through downregulating and upregulating the expression of *FUBP1* and *RAB2A,* respectively. These results led us to identify FUBP1 and RAB2A as susceptible genes for the hospitalization of COVID-19 and to reason that FUBP1 played an anti-viral role while RAB2A played a pro-viral role in the infection of SARS-CoV-2.

FUBP1 is a canonical RBP that belongs to a conserved family of single-stranded (ss) DNA-binding regulators named FUBPs^58^. Although FUBP1 is primarily located in the nucleus, reports suggest it can also be expressed in the cytoplasm, where it regulates cytoplasmic virus RNA^59^. Researchers found that *FUBP1* was downregulated significantly in COVID-19 patients compared to healthy controls in peripheral blood monocytes^60^, consistent with our finding in lung tissue. Although little is known about the function of FUBP1 in COVID-19, evidence has shown that FUBP1 suppresses protein translation of Japanese encephalitis virus (JEV) by targeting its 5’ and 3’ UTR^61^. Since FUBP1 binds to SARS-CoV-2, we propose that FUBP1 functions in an anti-viral way by suppressing transcription or protein translation. It has been validated that FUBP1 binds to the negative-sense of 29534-29870 in SARS-CoV-2 RNA (ORF10, 3’UTR, and poly(A)) in Calu-3 cells and might play a transcription role^62^. We speculate that FUBP1 functions in an anti-viral way by binding to ORF10 and 3’UTR of the negative strand in SARS-CoV-2 RNA and repressing the transcription of SARS-CoV-2. It is promising to target FUBP1 in the treatment of COVID-19 since a study has found that FUBP1 was repressed after SARS-CoV-2 infection in Calu-3 cells, and expression of FUBP1 could be reversed after allicin exposure to SARS-CoV-2-infected cells^63^.

RAB2A is a Rab family member and was first identified as a novel RBP in 2018^64^. Though little is known about its function on virus RNA, the role of RAB2A has been well illustrated as a critical modulator of intracellular membrane trafficking, especially in protein transport in ER–Golgi intermediate compartment (ERGIC)^65,66^. Knockdown of *RAB2A* induces Golgi fragmentation in HeLa-S3 cells^65^. RAB2A may play an essential role in ERGIC, where structural and non-structural proteins are assembled and transported to the cell surface and other organelles^9,67^. SARS-CoVs have been reported to exploit the intermediate compartment (IC) as an intracellular site of formation^68^. SARS-CoV-2 may hijack the host early secretory pathway involving RAB2A to assemble and transport. Moreover, RAB2A plays a vital role in the fusion of lysosomes (LYSs) and late endosomes (LEs)^69^. It has been found that RAB2A interacts with NSP7 and ORF3a^70,71^. ORF3a may bind to RAB2A and inhibit the fusion of LEs and LYSs through interaction with RAB2A, thus impairing the formation of autophagolysosomes. Moreover, a CRISPR screen in Huh-7.5 cells infected with SARS-CoV-2 showed that RAB2A was critical for virus replication and virus-induced cytopathic effect (CPE), indicating its significance in virus replication^70^. Considering that RAB2A has been identified as a SARS-CoV-2 binding protein, we put forward another assumption that SARS-CoV-2 hijacks RAB2A to facilitate the replication of its genome. Recent studies have shown that RAB2A interacts with NSP7 of SARS-CoV-2^70^, which is an indispensable co-factor binding to NSP12 to form an RdRp complex and plays a vital role in the stabilization of NSP12 regions involved in RNA binding^72^. We reason that RAB2A might contribute to stabilizing SARS-CoV-2 RNA by forming a complex where RAB2A binds to both vRNA and NSP7, thus facilitating the replication of SARS-CoV-2. Consistent with our study, a meta-analysis of GWAS revealed that RAB2A (rs13276831) was associated with severe COVID-19, and more expression of *RAB2A* was associated with worse disease^73^. In addition, a Mendelian randomization analysis based on transcriptome‐wide summary data associated *RAB2A* with hospitalized COVID-19 in Artery Tibial^74^. Strong evidence from genetic variants near protein-encoding loci (cis-pQTL) analysis combined with GWAS also suggested that *RAB2A* was a possible causal gene for severe COVID-19^75,76^. These studies verify the results of our research. Searching for the ChEMBL database, we identify CID1067700 as a promising inhibitor of RAB2A, which has also been shown to block Arf-like small GTPase Arl8b that regulates SARS-CoV-2 egress^77^. Further experiments are required to explore the effectiveness of CID1067700 in targeting RAB2A in treating COVID-19.

Even though few potential drugs have been found to target FUBP1 and RAB2A, using RNA-binding proteins to treat COVID-19 is still promising. Researchers have found pioglitazone and lapatinib as potential drugs since pioglitazone decreases the expression of RPS3, eIF4B, and RPS10, while lapatinib decreases the expression of EEF1A1, EIF5A, and RPS10 RBPs^78^. Another study investigated drugs targeting the interacting proteins of SARS-CoV-2-related RBPs and identified Doxorubicin and Topotecan as possible drugs targeting protein interactome of RBPs^79^, providing another way to use RBPs in the treatment of COVID-19.

Our data mining study identified two RBPs as susceptible genes for hospitalization of COVID-19. However, we failed to identify the exact binding sites of RAB2A on SARS-CoV-2 RNA due to limited CLIP-seq data. Further investigations, including RNA Binding Protein Immunoprecipitation Assay Sequencing (RIP-seq) and Gel Shift Assays (EMSA) are warranted to determine the exact binding sites of FUBP1 and RAB2A on SARS-CoV-2 thus to analyze the effects of variations of SARS-CoV-2 RNA on the binding sites. We also did not explain the further mechanism by which NC_000001.11:g.77984833C>A and NC_000008.11:g.60559280T>C regulate the expression level of *FUBP1* and *RAB2A*, respectively. CRISPR-mediated inhibition (CRISPRi) and activation (CRISPRa) could be used to validate the genetic effects of these two variants on RBPs, and ChIP-Seq (or ChIP-qPCR) could investigate the binding affinity of TFs. It remains unclear how FUBP1 and RAB2A function in SARS-CoV-2 infection. Further studies such as CRISPR are needed to clarify in which process the anti-viral role of FUBP1 and the pro-viral role of RAB2A play in COVID-19.

**Figure 6:**
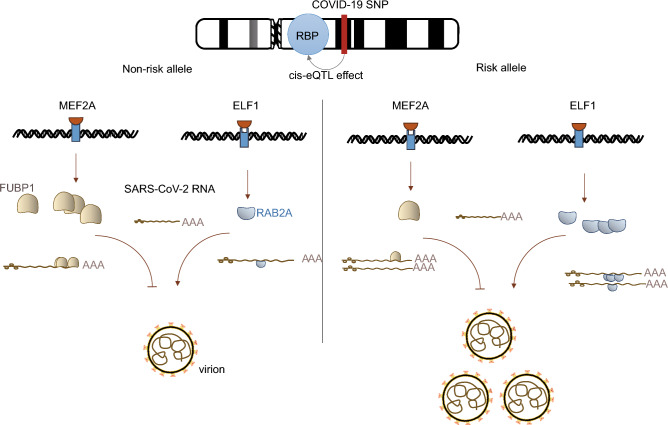
Hypothesis model of risk variants mapped to RBPs predispose hospitalization of COVID-19. MEF2A strongly binds to the non-risk allele of FUBP1 and ELF1 weakly binds to that of RAB2A, which enhances the anti-viral ability of FUBP1 and reduces the pro-viral capacity of RAB2A, leading to less virion. In contrast, MEF2A weakly binds to the risk allele of FUBP1, and ELF1 strongly binds to that of RAB2A, which reduces the anti-viral ability of FUBP1 and enhances the pro-viral capacity of RAB2A, leading to more virion and worse outcome of COVID-19.

In summary, this study annotates risk genes from genetic variants by combining variant function and cis-eQTL and delves into the interaction between host RBPs and SARS-CoV-2. This study identifies two key contributing genes, *FUBP1* and *RAB2A*, whose corresponding proteins bind to the genome of SARS-CoV-2 in SARS-CoV-2-infected epithelial cells in the lung. This study also highlights that two functional variants regulate the expression of these two RBPs by altering the binding affinity of TFs, indicating the anti-viral role of FUBP1 and the pro-viral role of RAB2A. This study provides valuable insights into the genetic risk genes in the pathogenesis of SARS-CoV-2 and provides compelling evidence for further investigations into the role of FUBP1 and RAB2A as RBPs in SARS-CoV-2 infection. The study proposes a model that suggests host variants may change the TF binding affinity, thereby altering the expression levels of RBPs and influencing the function of RBPs interacting with the virus (Fig. 6). The findings offer novel perspectives in the realm of RNA virus research, particularly with regards to the interaction between host RBPs and viral RNA.

### Supplementary Information

Supplementary Information 1.

Supplementary Information 2.

## Data Availability

All data and materials related to the study are publicly available. The data that support the findings of this study are openly available in the COVID-19 Host Genetics Initiative at https://www.covid19hg.org/^24^; Genotype-Tissue Expression Portal at https://gtexportal.org/home/^28^; Single Cell Portal at https://singlecell.broadinstitute.org/single_cell/study/SCP1289/; cellxgene at https://cellxgene.cziscience.com/collections/6f6d381a-7701-4781-935c-db10d30de293; ENCODE portal at https://www.encodeproject.org/^44^.
