# Supplementary material for: Integration of risk variants from GWAS with SARS-CoV-2 RNA interactome prioritizes FUBP1 and RAB2A as risk genes for COVID-19
Source: Sci Rep. 2023 Nov 6;13:19194. doi: 10.1038/s41598-023-44705-3 (PMC10628159; doi:10.1038/s41598-023-44705-3)
Supplement: Supplementary file 1 — Supplementary Information 1. [file 41598_2023_44705_MOESM1_ESM.zip › Supplementary Figures.pdf]

Integration of risk variants from GWAS with SARS-CoV-2 RNA interactome prioritizes FUBP1 and RAB2A as risk genes for COVID-19

Weiwen Shi [1, +], Mengke Chen [1, +], Tingting Pan [1], Mengjie Chen [3], Yongjun Cheng [3], Sheng Chen [1], and Yuanjia Tang [1,2, *]

1 Shanghai Institute of Rheumatology/Department of Rheumatology, Renji Hospital, Shanghai Jiao Tong University School of Medicine, Shanghai, China; yjtang@sibs.ac.cn
2 State Key Laboratory of Oncogenes and Related Genes, Shanghai Cancer Institute, Renji Hospital , Shanghai, China; yjtang@sibs.ac.cn
3 Department of Rheumatology, the First People' s Hospital of Wenling

* Correspondence: yjtang@sibs.ac.cn
+ These authors contributed equally to this work.

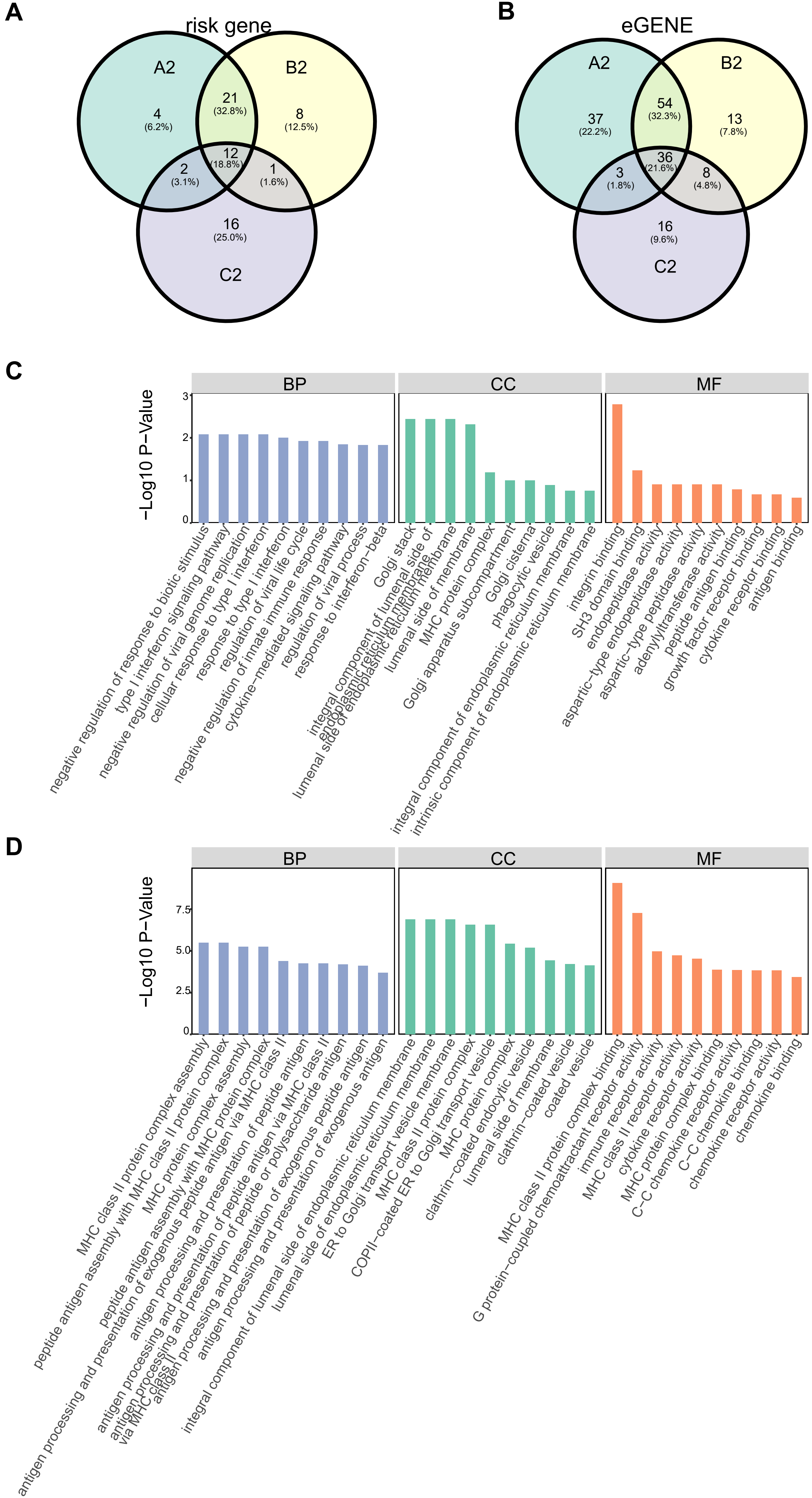

A
risk gene
A2
B2
C2
4
(6.2%)
21
(32.8%)
8
(12.5%)
12
(18.8%)
2
(3.1%)
1
(1.6%)
16
(25.0%)
B
eGENE
A2
B2
C2
37
(22.2%)
54
(32.3%)
13
(7.8%)
36
(21.6%)
3
(1.8%)
8
(4.8%)
16
(9.6%)
C
BP
CC
MF
−Log10 P−Value
0
1
2
3
negative regulation of response to biotic stimulus
type I interferon signaling pathway
negative regulation of viral genome replication
cellular response to type I interferon
response to type I interferon
regulation of viral life cycle
negative regulation of innate immune response
cytokine−mediated signaling pathway
regulation of viral process
response to interferon−beta
Golgi stack
integral component of lumenal side of endoplasmic reticulum membrane
lumenal side of endoplasmic reticulum membrane
lumenal side of membrane
MHC protein complex
Golgi apparatus subcompartment
Golgi cisterna
phagocytic vesicle
integral component of endoplasmic reticulum membrane
intrinsic component of endoplasmic reticulum membrane
integrin binding
SH3 domain binding
endopeptidase activity
aspartic−type endopeptidase activity
aspartic−type peptidase activity
adenylyltransferase activity
peptide antigen binding
growth factor receptor binding
cytokine receptor binding
antigen binding
D
BP
CC
MF
−Log10 P−Value
0
2.5
5.0
7.5
MHC class II protein complex assembly
peptide antigen assembly with MHC class II protein complex
MHC protein complex assembly
peptide antigen assembly with MHC protein complex
antigen processing and presentation of exogenous peptide antigen via MHC class II
antigen processing and presentation of peptide antigen
antigen processing and presentation of peptide antigen via MHC class II
antigen processing and presentation of peptide or polysaccharide antigen via MHC class II
antigen processing and presentation of exogenous peptide antigen
antigen processing and presentation of exogenous antigen
integral component of lumenal side of endoplasmic reticulum membrane
lumenal side of endoplasmic reticulum membrane
ER to Golgi transport vesicle membrane
MHC class II protein complex
COPII−coated ER to Golgi transport vesicle
MHC protein complex
clathrin−coated endocytic vesicle
lumenal side of membrane
clathrin−coated vesicle
coated vesicle
MHC class II protein complex binding
G protein−coupled chemoattractant receptor activity
immune receptor activity
MHC class II receptor activity
cytokine receptor activity
MHC protein complex binding
C−C chemokine receptor activity
C−C chemokine binding
chemokine receptor activity
chemokine binding

Supplementary Figure S1 Mapping risk genes associated with COVID-19 GWAS SNPs Vennplot of (A) genes with nonsynonymous substitutions and (B) eGENEs for COVID-19 in A2, B2 and C2 phenotype. Barplot of enriched GO Pathways of (C) genes with nonsynonymous substitutions and (D) eGENEs for COVID-19.

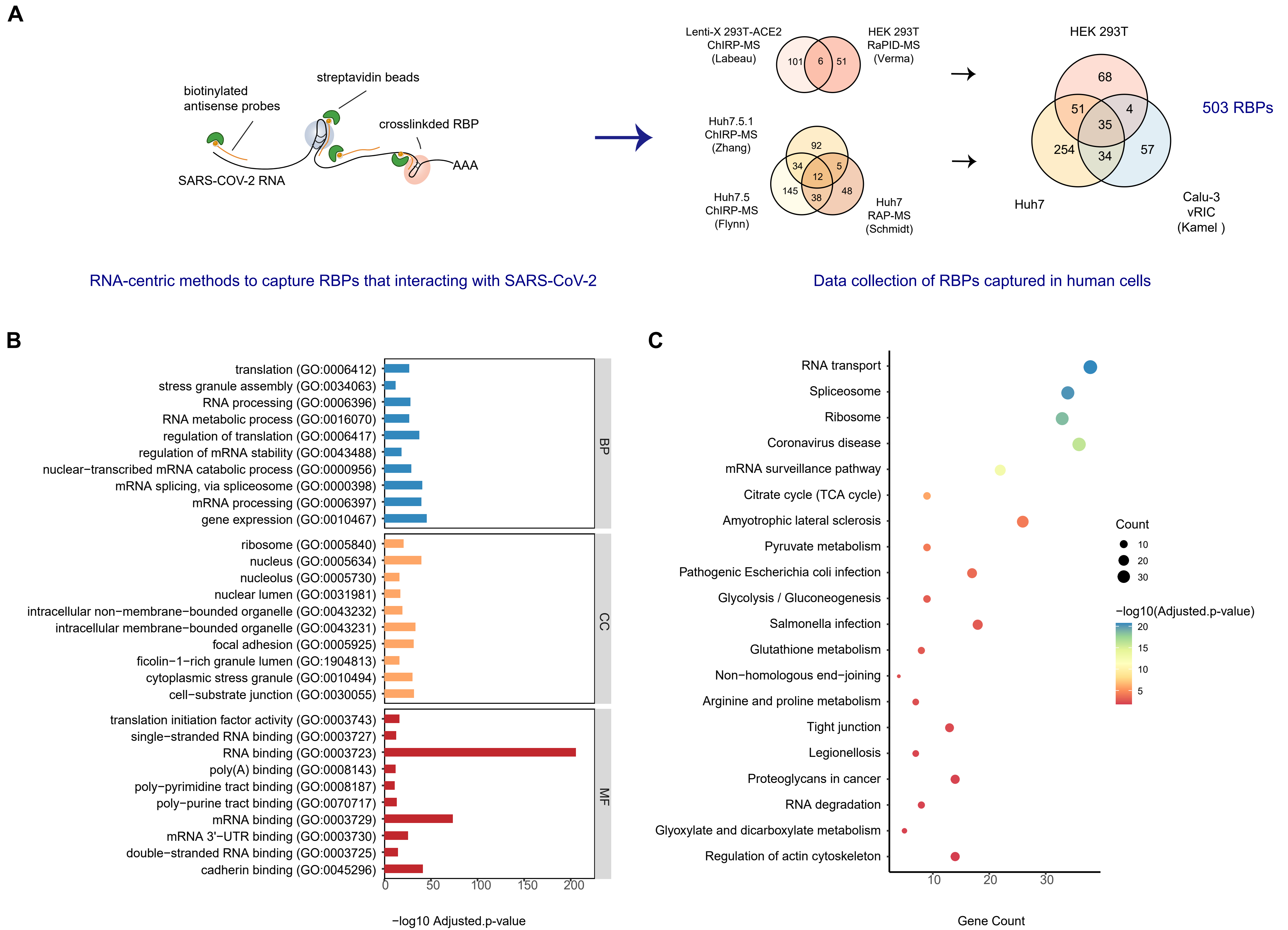
A
biotinylated antisense probes
streptavidin beads
crosslinkded RBP
SARS-COV-2 RNA
AAA
RNA-centric methods to capture RBPs that interacting with SARS-CoV-2
Lenti-X 293T-ACE2 ChIRP-MS (Labeau)
HEK 293T RaPID-MS (Verma)
101
6
51
Huh7.5.1 ChIRP-MS (Zhang)
Huh7.5 ChIRP-MS (Flynn)
Huh7 RAP-MS (Schmidt)
92
34
5
12
145
38
48
HEK 293T
Huh7
Calu-3 vRIC (Kamel)
68
51
4
35
254
34
57
503 RBPs
Data collection of RBPs captured in human cells
B
translation (GO:0006412)
stress granule assembly (GO:0034063)
RNA processing (GO:0006396)
RNA metabolic process (GO:0016070)
regulation of translation (GO:0006417)
regulation of mRNA stability (GO:0043488)
nuclear−transcribed mRNA catabolic process (GO:0000956)
mRNA splicing, via spliceosome (GO:0000398)
mRNA processing (GO:0006397)
gene expression (GO:0010467)
ribosome (GO:0005840)
nucleus (GO:0005634)
nucleolus (GO:0005730)
nuclear lumen (GO:0031981)
intracellular non−membrane−bounded organelle (GO:0043232)
intracellular membrane−bounded organelle (GO:0043231)
focal adhesion (GO:0005925)
ficolin−1−rich granule lumen (GO:1904813)
cytoplasmic stress granule (GO:0010494)
cell−substrate junction (GO:0030055)
translation initiation factor activity (GO:0003743)
single−stranded RNA binding (GO:0003727)
RNA binding (GO:0003723)
poly(A) binding (GO:0008143)
poly−pyrimidine tract binding (GO:0008187)
poly−purine tract binding (GO:0070717)
mRNA binding (GO:0003729)
mRNA 3'−UTR binding (GO:0003730)
double−stranded RNA binding (GO:0003725)
cadherin binding (GO:0045296)
BP
CC
MF
0
50
100
150
200
−log10 Adjusted.p-value
C
RNA transport
Spliceosome
Ribosome
Coronavirus disease
mRNA surveillance pathway
Citrate cycle (TCA cycle)
Amyotrophic lateral sclerosis
Pyruvate metabolism
Pathogenic Escherichia coli infection
Glycolysis / Gluconeogenesis
Salmonella infection
Glutathione metabolism
Non−homologous end−joining
Arginine and proline metabolism
Tight junction
Legionellosis
Proteoglycans in cancer
RNA degradation
Glyoxylate and dicarboxylate metabolism
Regulation of actin cytoskeleton
10
20
30
Gene Count
Count
10
20
30
−log10(Adjusted.p-value)
20
15
10
5

Supplementary Figure S2 RBPs identified in RNA-centric screens for SARS-CoV-2 (A) The number of proteins identified in SARS-CoV-2 RNA interactome in human cell lines. (B) Barplot of enriched GO Pathways. (C) Dotplot of top 20 Enriched KEGG Pathways.

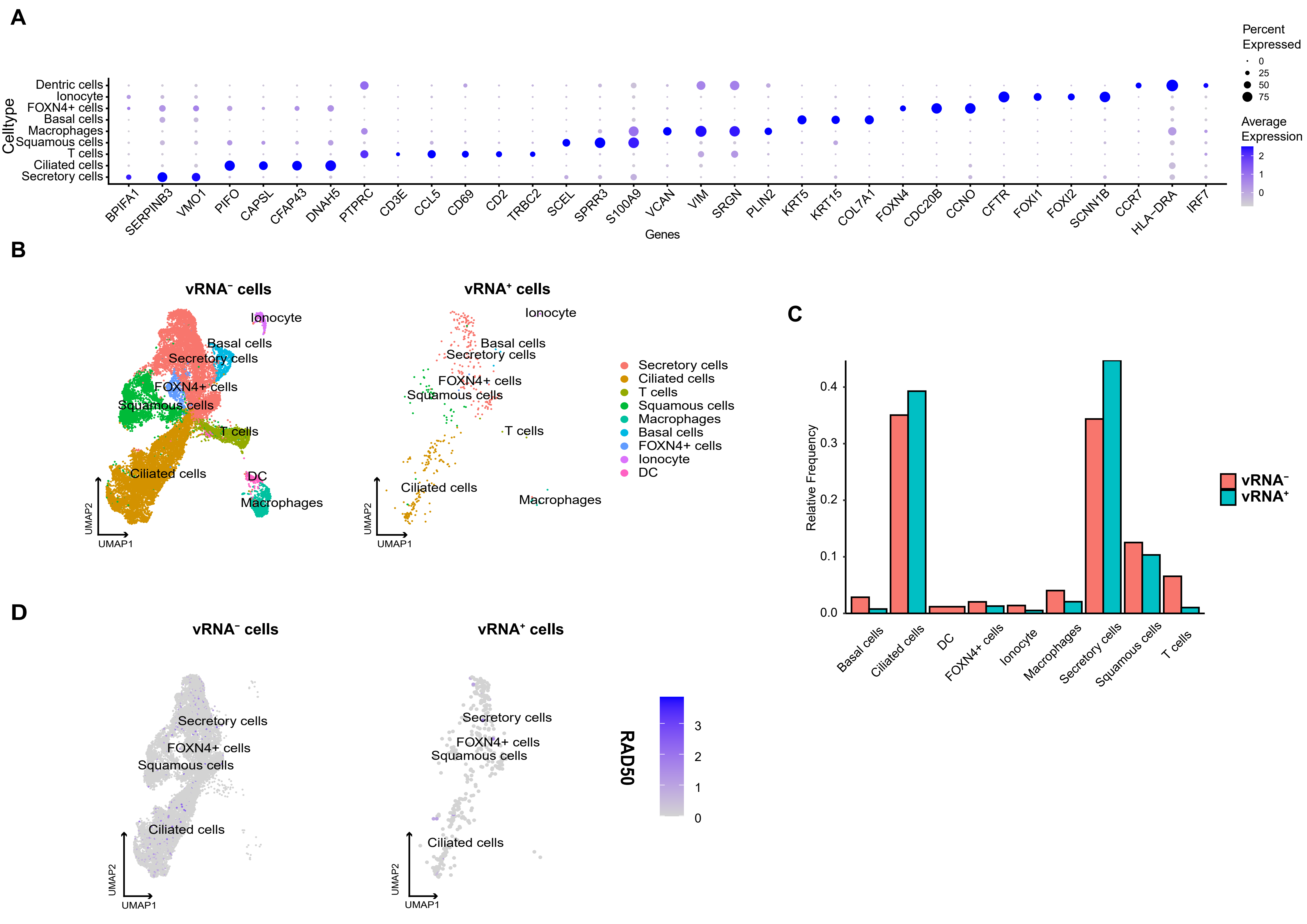
A
Percent Expressed
0
25
50
75
Average Expression
2
1
0
Celltype
Dentric cells
Ionocyte
FOXN4+ cells
Basal cells
Macrophages
Squamous cells
T cells
Ciliated cells
Secretory cells
BPIFA1
SERPINB3
VMO1
PIFO
CAPSL
CFAP43
DNAH5
PTPRC
CD3E
CCL5
CD69
CD2
TRBC2
SCEL
SPRR3
S100A9
VCAN
VIM
SRGN
PLIN2
KRT5
KRT15
COL7A1
FOXN4
CDC20B
CCNO
CFTR
FOXI1
FOXI2
SCNN1B
CCR7
HLA-DRA
IRF7
Genes
B
vRNA⁻ cells
vRNA⁺ cells
Ionocyte
Basal cells
Secretory cells
FOXN4+ cells
Squamous cells
T cells
Ciliated cells
DC
Macrophages
UMAP2
UMAP1
Secretory cells
Ciliated cells
T cells
Squamous cells
Macrophages
Basal cells
FOXN4+ cells
Ionocyte
DC
C
Relative Frequency
0.0
0.1
0.2
0.3
0.4
vRNA⁻
vRNA⁺
Basal cells
Ciliated cells
DC
FOXN4+ cells
Ionocyte
Macrophages
Secretory cells
Squamous cells
T cells
D
vRNA⁻ cells
vRNA⁺ cells
Secretory cells
FOXN4+ cells
Squamous cells
Ciliated cells
RAD50
3
2
1
0
UMAP2
UMAP1

Supplementary Figure S3 Summary of scRNA-seq data (A) Dotplot of cell-specific expression of marker genes for single cell annotation. (B) UMAP representation of single-cell RNA sequencing data with annotations of cell types. (C) Barplot showing the relative frequency of annotated cells in $vRNA^{+}$ and $vRNA^{-}$ groups. (D) The feature plots showing the expression levels of RAD50 in the four epithelial cells, which are grouped by SARS-CoV-2 RNA status.

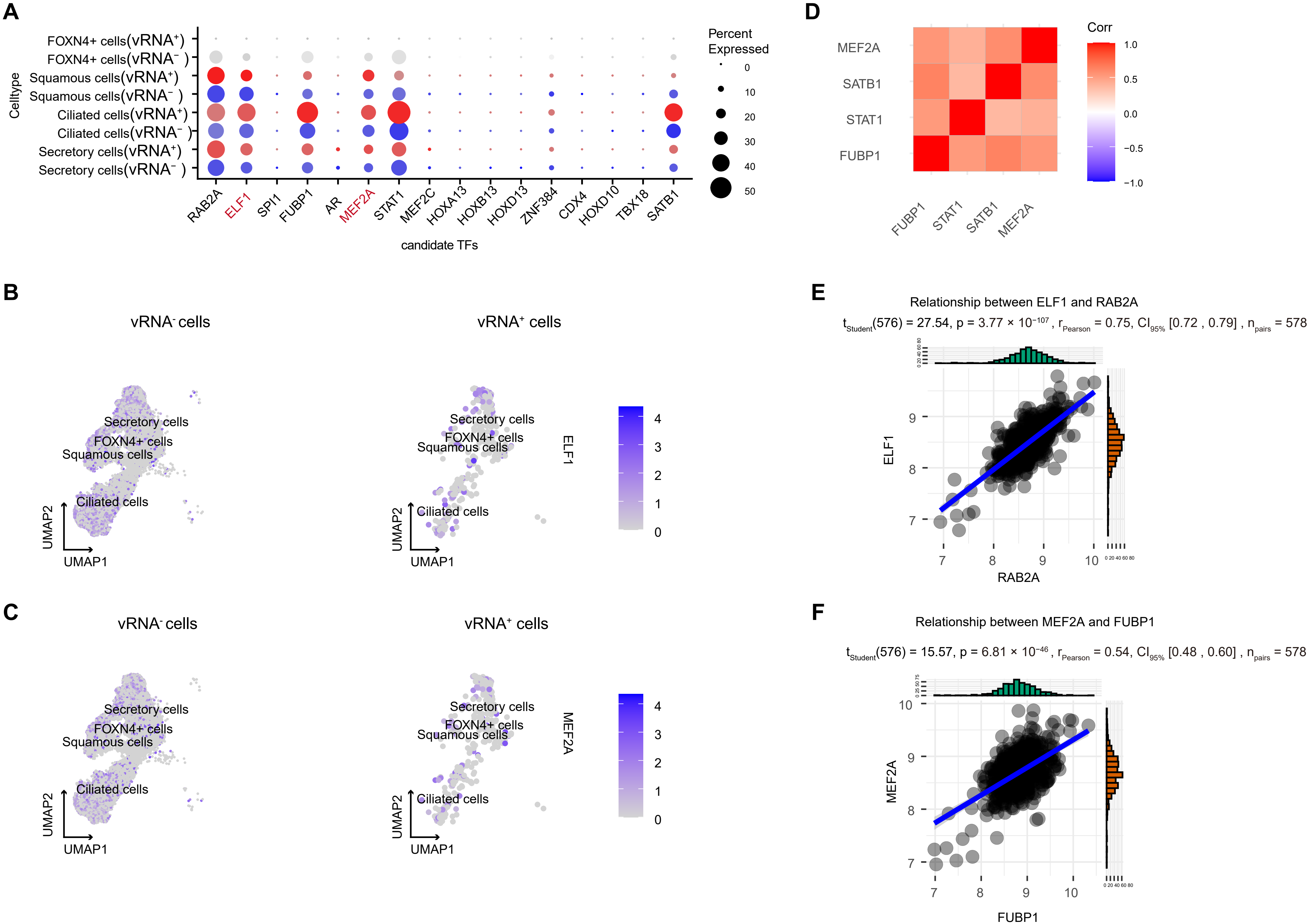
A
FOXN4+ cells(vRNA⁺)
FOXN4+ cells(vRNA⁻)
Squamous cells(vRNA⁺)
Squamous cells(vRNA⁻)
Ciliated cells(vRNA⁺)
Ciliated cells(vRNA⁻)
Secretory cells(vRNA⁺)
Secretory cells(vRNA⁻)
Celltype
RAB2A
ELF1
SPI1
FUBP1
AR
MEF2A
STAT1
MEF2C
HOXA13
HOXB13
HOXD13
ZNF384
CDX4
HOXD10
TBX18
SATB1
candidate TFs
Percent Expressed
0
10
20
30
40
50
D
MEF2A
SATB1
STAT1
FUBP1
FUBP1
STAT1
SATB1
MEF2A
Corr
1.0
0.5
0.0
−0.5
−1.0
B
vRNA⁻ cells
vRNA⁺ cells
Secretory cells
FOXN4+ cells
Squamous cells
Ciliated cells
UMAP2
UMAP1
ELF1
4
3
2
1
0
E
Relationship between ELF1 and RAB2A
$t_{Student}(576) = 27.54$, $p = 3.77 \times 10^{-107}$, $r_{Pearson} = 0.75$, $CI_{95\%}$ [0.72 , 0.79] , $n_{pairs} = 578$
ELF1
RAB2A
7
8
9
10
C
vRNA⁻ cells
vRNA⁺ cells
Secretory cells
FOXN4+ cells
Squamous cells
Ciliated cells
UMAP2
UMAP1
MEF2A
4
3
2
1
0
F
Relationship between MEF2A and FUBP1
$t_{Student}(576) = 15.57$, $p = 6.81 \times 10^{-46}$, $r_{Pearson} = 0.54$, $CI_{95\%}$ [0.48 , 0.60] , $n_{pairs} = 578$
MEF2A
FUBP1
7
8
9
10

Supplementary Figure S4 Expression of candidate TFs in single-cell RNA sequencing data and relationship between TFs and RBPs Dotplot showing the number of cells expressing candidate Transcription factors in both vRNA+ and vRNA? groups. (B) heatmap showing the corrleation between RBPs and candidate TFs. Featureplots show expression levels of (B) ELF1, (C) MEF2A in scRNA-seq data grouped by SARS-CoV-2 RNA status. Corrrlationplot showing relation ship between (E)ELF1 and RAB2A and (F) MEF2A and FUBP1.
