# Supplementary material for: Integration of risk variants from GWAS with SARS-CoV-2 RNA interactome prioritizes FUBP1 and RAB2A as risk genes for COVID-19
Source: Sci Rep. 2023 Nov 6;13:19194. doi: 10.1038/s41598-023-44705-3 (PMC10628159; doi:10.1038/s41598-023-44705-3)
Supplement: Supplementary file 1 — Supplementary Information 1. [file 41598_2023_44705_MOESM1_ESM.zip › Supplementary Materials.docx]

**Supplementary Materials**

Table S1: Annotation of all significant variants for COVID-19 and those variants in high LD with them (R^2^ >0.8, EUR pop).

Table S2: GO enrichment of risk genes of COVID-19.

Table S3: A collection of SARS-CoV-2 RNA interactome using multiple RNA-centric methods in different cell lines.

Table S4: Candidate allelic-specific TFs in NC_000008.11:g.60559280T>C.

Table S5: TFs predicted to bind in NC_000001.11:g.77984833C>A.
